# Impact of chitin nanofibers and nanocrystals from waste shrimp shells on mechanical properties, setting time, and late-age hydration of mortar

**DOI:** 10.1038/s41598-022-24366-4

**Published:** 2022-11-29

**Authors:** Md. Mostofa Haider, Guoqing Jian, Hui Li, Quin R. S. Miller, Michael Wolcott, Carlos Fernandez, Somayeh Nassiri

**Affiliations:** 1 Department of Civil and Environmental Engineering, Washington State University, Pullman, WA 99163 USA; 2 Pacific Northwest National Laboratory, Richland, WA 99352 USA; 3 Composite Materials and Engineering Center, Washington State University, Pullman, WA 99163 USA; 4 Department of Civil and Environmental Engineering, Washington State University, Pullman, WA 99164 USA

**Keywords:** Civil engineering, Nanoscale materials

## Abstract

Every year ~ 6–8 million tonnes of shrimp, crab, and lobster shell wastes are generated, requiring costly disposal procedures. In this study, the chitin content of shrimp shell waste was oxidized to produce chitin nanocrystals (ChNC) and mechanically fibrillated to obtain chitin nanofibers (ChNF) and evaluated as additives for mortar. ChNF (0.075 wt%) and ChNC (0.05 wt%) retarded the final setting time by 50 and 30 min, likely through cement dispersion by electrostatic repulsion. ChNF (0.05 wt%) with a larger aspect ratio than ChNC resulted in the greatest improved flexural strength and fracture energy by 24% and 28%. Elastic modulus increased by up to 91% and 43% with ChNC and ChNF. Solid-state nuclear magnetic resonance (NMR) showed ChNF (0.05 wt%) enhanced calcium–silicate–hydrate structure with a 41% higher degree of polymerization, 9% more silicate chain length, and a 15% higher degree of hydration at 28 days. Based on the findings, chitin seems a viable biomass source for powerful structural nanofibers and nanocrystals for cementitious systems to divert seafood waste from landfills or the sea.

## Introduction

Gigatons of concrete are used yearly to build residential and industrial buildings and a myriad of infrastructures essential for societies' livelihood, health, and safety. The versatility of concrete stems in its unique ability to be made with local aggregates poured in any form and shape and develop high strength within hours. However, manufacturing this popular construction material consumes 2/5 of natural stone/sand/aggregate reserves, 1/5 of virgin woods, 9% of industrial water withdrawal^1^, and 12–15% of industrial energy^2,3^. Furthermore, concrete production is responsible for 8% of anthropogenic CO_2_ emissions^4^. Portland Cement Association (PCA) 's recent roadmap to carbon neutrality alludes to decreasing maintenance in concrete infrastructure as a means to reduce emissions^5^. Therefore, sustainable solutions are urgently needed to address the issue of brittleness and cracking in concrete to curb infrastructure repair needs^6^. Because concrete fracture is a multiscale phenomenon, various size fibers have been explored over the years to arrest cracks and bridge pores in the relevant scale^6–8^. Recent advances in nanomaterials production at pilot and full scales have made it possible to consider nano-reinforced concrete. Nanofibers have the tremendous advantage of superior performance at a minimal fraction dosage of macro and microfibers^9^. In addition, biobased nanomaterials have the advantages of renewability and nontoxicity of the source material over synthetic counterparts. Furthermore, using biomass wastes as source materials diverts wastes from the environment, closes the materials loop, and promotes a circular economy.

In this study, we repurpose non-traditional biomass waste as value-added nano-reinforcement for cement-based materials. Our source material is chitin, the second most abundant natural polymer, produced on a 100 billion tons scale in various living organisms. Chitin is present in the exoskeleton of shellfish (crab, shrimp, lobster, etc.), insects, and some other plants/animals^10^. The seafood industry's estimated 6–8 million tons of yearly waste is about 70% bark and contains 20–30% chitin. Most of this bark is composted or discarded in landfills or the ocean. Disposal of this waste in the ocean without treatment harms the aquatic species and causes coastal pollution^11^. Chitin and its primary derivative, chitosan, have been studied as a biopolymer admixture in cementitious materials to modify the rheology (viscosity) and setting time, retain heavy metals, and inhibit rebar corrosion^12–15^. However, the significant structural benefits of the nanofiber constituents of chitin and their potential ability to reinforce cement at the nanoscale have been overlooked. Chitin has semicrystalline structures from which chitin nanomaterials (ChNMs) can be extracted with a high degree of crystallinity and excellent mechanical properties [modulus ~ 41 GPa,^16^], and extremely high specific surface area of 173–350 m^2^/g^17,18^. Furthermore, the hydroxyl and amide groups in the polymer structure of chitin can form hydrogen bonds with the host material to render a strong reinforced composite performance.

ChNMs can be made in rod-like nanocrystal (ChNC) or nanofiber (ChNF) morphologies using chemical or chemo-mechanical extraction processes^19^. The closest comparisons to rod-like or fibrillar ChNMs are cellulose nanocrystals (CL-NC) and cellulose nanofibers (CL-NF) derived from different cellulosic sources by various methods^20,21^. Previous studies found that cellulose-based nanomaterials influence cementitious systems' rheological and mechanical properties, with mostly positive outcomes^22–25^. Most studies reported an increased degree of hydration (DOH) with CL-NC and CL-NF, resulting in improved strength^9,25–27^. CL-NF has a highly reactive surface with hydroxyl groups that facilitate high hydrogen bonding with the cementitious matrix^28^. CL-NFs have a high surface-area-to-volume ratio, high modulus [65–145 GPa^29^], high tensile strength, and large aspect ratio [25–500]^20^, resulting in improved mechanical and microstructural performance in the cementitious system. As chitin has a similar structure to cellulose, a source of high-performance nano-derivatives with great demonstrated benefits for cementitious systems, ChNMs also deserve investigation for reinforcement of cement-based systems. However, the potential of ChNMs has not been explored for the general use cement paste/mortar/concrete previously.

Therefore, this study is focused on the performances of two different ChNMs; TEMPO-oxidized ChNC and mechanically fibrillated ChNF derived from raw chitin from shrimp shell waste. The two ChNMs are characterized by transmission electron microscope (TEM) imaging, x-ray diffractometer (XRD), Fourier-transform infrared spectroscopy (FTIR), zeta potential, and conductometric titration. Next, the properties of mortar with the two different ChNMs in various dosages are presented, focusing on setting time, mechanical properties, and late-age hydration. For mechanical properties, compressive strength, modulus of elasticity, flexural strength, and fracture energy are presented and discussed. To further understand the mechanism of the effect of ChNMs on strength development and late-age hydration, total organic carbon (TOC), XRD, solid-state nuclear magnetic resonance (NMR), FTIR, and thermogravimetric analysis (TGA) of mortar was performed on cement paste samples, and the interrelationships of test results are fully discussed.

## Materials and methods

### Production of chitin nanomaterials

**Figure 1:**
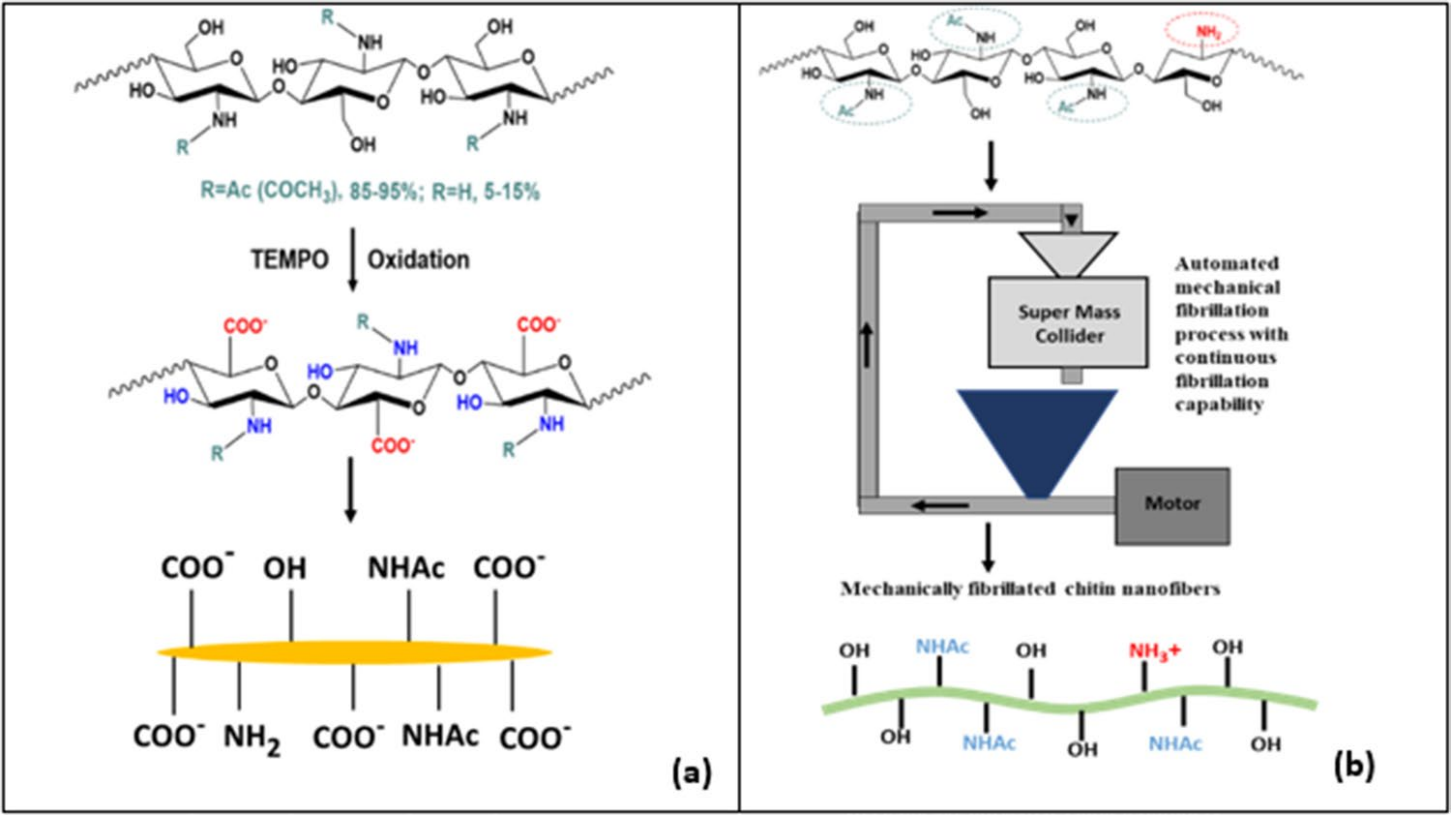
Schematics of production of ChNMs by (**a**) TEMPO-oxidation (ChNC), and (**b**) mechanical fibrillation with SMC (ChNF).

Shrimp shell-derived chitin powder (Sigma Aldrich) was used to produce ChNMs. The TEMPO-mediated oxidation process produced chitin nanocrystals (ChNCs). In the TEMPO-mediated oxidation process, the OH group at the C-6 position is selectively oxidized to the COO^−^ group. The schematic of the process is shown in Fig. 1a. The authors described more details of the TEMPO-mediated oxidation process in another publication^30^. The final ChNC products were suspensions with an approximately 1% solid weight concentration. The apparent density of the ChNM suspensions was not characterized in this study but is expected to be similar to that of cellulose NF at 1.6 g/cm^3^ reported in another study^31^.

Chitin nanofibers (ChNF) were produced in a mechanical process from raw chitin using a grinding disc setup that applies significant compression, shearing, and friction forces using a Super Mass Colloider (SMC) (Model MKCA6-5J, Masuko, Japan) as shown in Fig. 1b. The resultant ChNF suspension had a solids content of 0.82%. Since no chemical is used in this method, the fully mechanical process has little effect on the chemical structure of chitin. However, due to the higher specific surface area of the nanofibers, the density of the surface reactive groups, i.e., free hydroxyl groups and amine groups, are expected to be higher than ChNC.

### Characterization of nanochitins

Produced ChNMs were characterized for functional chemical groups, size, and morphological characteristics. Fourier-transform infrared (FTIR) spectroscopy was used to identify the functional groups of the ChNMs based on the intensity of the absorbed infrared radiation for a particular frequency directly associated with the vibrational footprint of the functional groups. Nicolet iS-50, Thermo Fisher Scientific, USA FTIR spectrometer was used to obtain the spectra of freeze-dried ChNMs under attenuated total reflection (ATR) mode for frequencies between 4000 and 600 cm^−1^ with a 4 cm^−1^ resolution and 64 scans.

Conductometric titration was performed to identify the concentration of surface functional groups of the ChNF and ChNC. In addition, the surface charge density of ChNM's was identified as described in the author's other works^30^.

Crystalline structure in ChNC and ChNF was determined using an X-ray diffractometer (XRD) (Rigaku Miniflex 600, Japan) with a CuKα X-ray source (λ = 0.1548 nm) at 40 kV and 15 mA. The crystallinity index was calculated by Eq. (1), where I_110_ is the intensity of the (110) plane diffraction peak, and I_AM_ represents the amorphous intensity of nanochitin at 2θ = 16.0°.

1 $$CrI=\frac{({I}_{110}-{I}_{AM})}{{I}_{110}}\times 100\%$$

The zeta potential (ς-potential) of ChNMs in dispersion media is important for understanding dispersion kinetics and electrostatic interactions with cement particles. Therefore, Zeta potential measurement was performed with a Malvern Zeta Analyzer on ChNC/ChNF suspensions at a concentration of 0.2 wt%.

The morphology of ChNM was observed with an FEI Tecnai G2 20 Twin TEM instrument (FEI company, USA) at 200 kV. The sample preparation was described in another reference^30^. Dimensions of the nanocrystals and the nanofibers were statistically quantified in terms of width (w), length (l), and aspect ratio (l/w) based on a ChNM sample size of n ≥ 100 using ImageJ software (NIH).

### Production of mortar formulations

**Figure 2:**
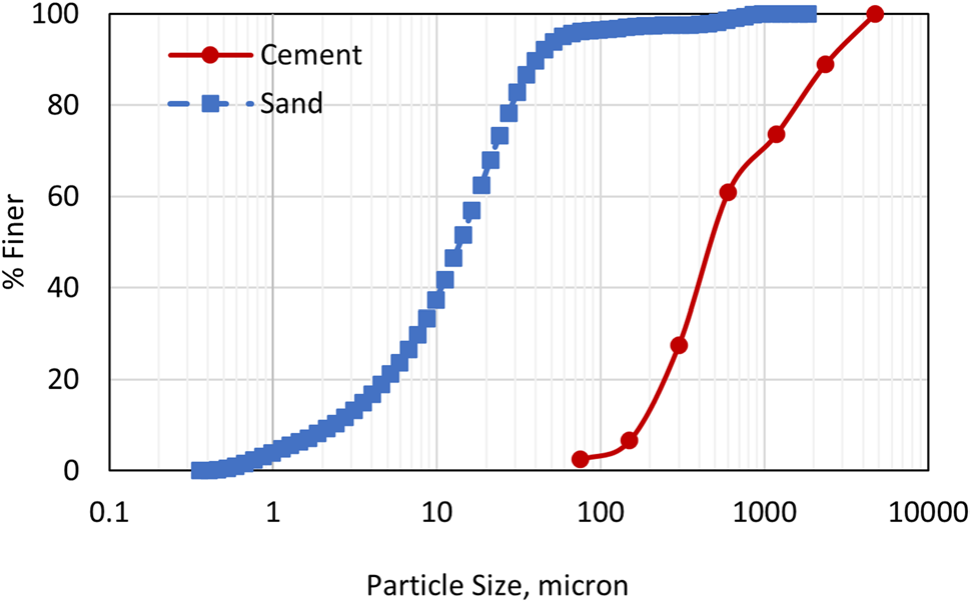
Particle size distribution of cement and sand used in mortar production.

ASTM C150 Type I-II Portland cement (PC) with the chemical composition and physical properties shown in Table 1 was used to produce all the mortars in this study. Natural sand with a dry specific gravity of 2.65, saturated surface dry (SSD) specific gravity of 2.69, and absorption of 1.75% was used. The particle size distribution of sand and PC is shown in Fig. 2.

**Table 1:** Chemical composition and physical properties of cement.

Chemical compound/property	%Mass
SiO_2_	21.3
Al_2_O_3_	3.2
Fe_2_O_3_	2.9
SO_3_	3.1
CaO	64.3
MgO	2.1
Na_2_O	0.26
K_2_O	0.42
Loss on ignition (wt%)	2.97
Blaine fineness (m^2^/kg)	391
Specific gravity	3.1

The proportions of the constituents of the control (baseline) mortar mixture were 1:2.5:0.52 by mass for cement:sand:water with zero wt% ChNM or any chemical admixtures. Different doses of ChNC and ChNF ranging from 0.02% to 0.1 wt% of dry cement were incorporated in the mortar. The proportions of the ChNM-mortars are shown in Table 2.

**Table 2:** Proportions of constituents of ChNC/ChNF-Mortar per 100 g of cement.

Mix name/ID	Cement (g)	Water (g)	ChNM solids/cement (wt%)	Sand
Control	100	52	0.000	250
0.020 ChNC	100	52	0.020	250
0.035 ChNC			0.035	
0.045 ChNC			0.045	
0.05 ChNC			0.050	
0.10 ChNC			0.100	
0.035 ChNF	100	52	0.035	250
0.045 ChNF			0.045	
0.050 ChNF			0.050	
0.055 ChNF			0.055	
0.065 ChNF			0.065	
0.10 ChNF			0.100	
0.05 ChNC + 0.05 ChNF	100	52	0.050 + 0.050	250
0.1SP + 0.1ChNC	100	52	0.100	
0.1SP + 0.1ChNF	100	52	0.100	

For ChNF, the loadings were 0.035%, 0.045%, 0,05%, 0.065%, 0.1% of cement weight. For ChNC, the loadings ranged from 0.02 to 0.1 wt% because our trials with ChNC and ChNF as well as cellulose NC and NF in our previous studies on the cement paste^25,32^ had shown that lower doses than ChNF work better for ChNC. Nanosized fillers typically work better in the cement matrix in small amounts compared to micro-sized fillers. The nanosized filler is usually used in less than 1% of the cement^33^, whereas micro-sized filler is used in a higher percentage, as seen for fly ash or silica fume. Nanosized fillers have a higher specific surface area compared to micro-sized fillers, which may provide more sites for nucleation, thus a better seed effect for improved hydration^34^. Furthermore, a higher aspect ratio, especially for ChNF, can provide better reinforcement at a low dose compared to the micro-sized filler.

In addition to these ChNM-mortars, one ChNM-mortar with a hybrid blend of ChNC and ChNF each at 0.05 wt% loading was included to learn about any synergic effect from these two ChNM on mechanical properties. Finally, two additional ChNM-mortars at the 0.1 wt% ChNC and ChNF were included with 0.1 wt% commercial carboxylate superplasticizer (SP) to identify any potential synergistic collaboration between ChNMs and a polymeric admixture that is commonly used in cement materials production.

## Mortar production procedure

**Figure 3:**
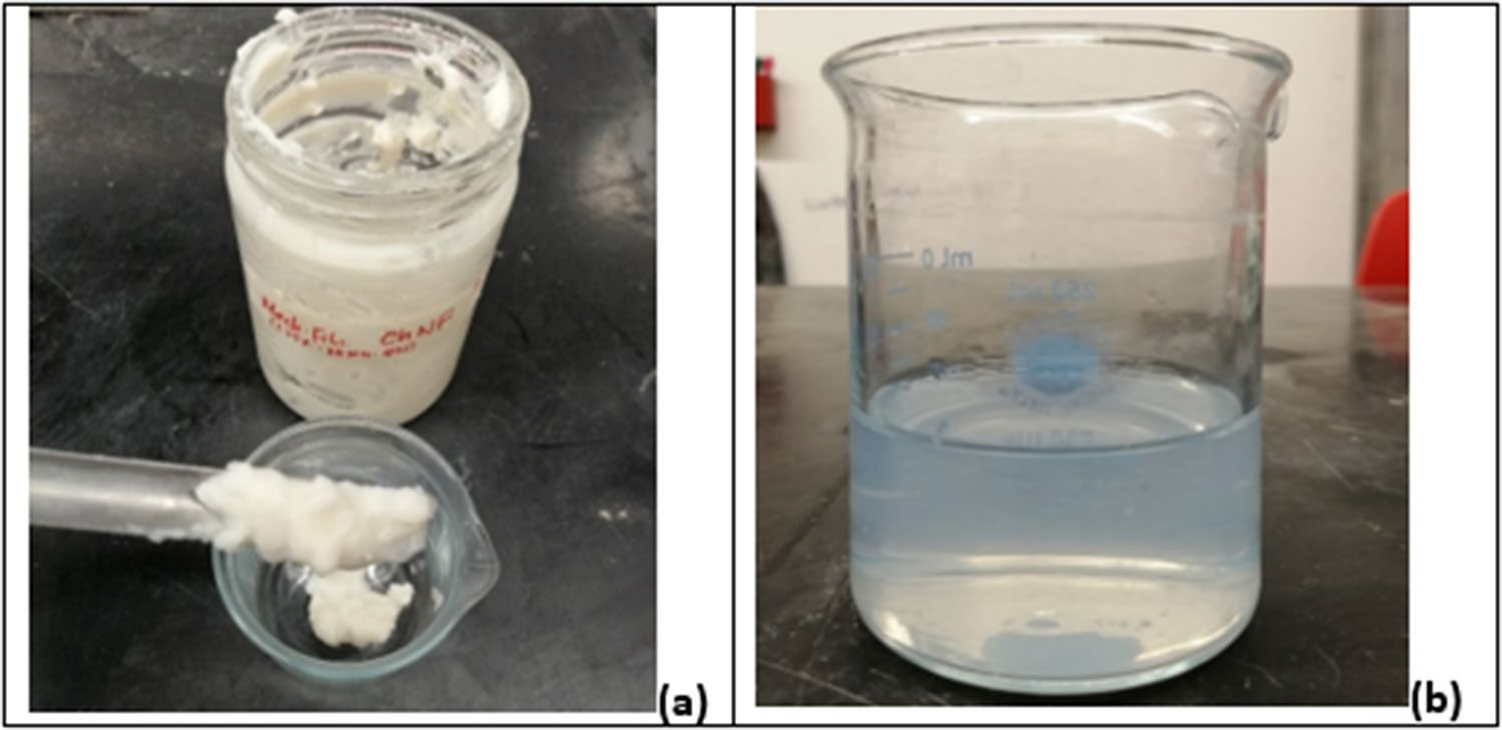
(**a**) ChNF being dispensed into the beaker, (**b**) ChNC before mixing into the mortar.

First, the desired ChNM slurry was taken in a beaker, and then 50 ml of water was added to dilute it and ease mixing (Fig. 3a). Afterward, to remove any potential agglomerates, ChNM suspensions were sonicated for 10 min before mixing with the cement (Fig. 3b).

The sonicated ChNM slurry was added to the remaining mixing water in the mixing bowl. The total mix water was adjusted based on the water content in ChNM slurry to keep the w/c content constant at 0.52. This slurry was then stirred for 1 min. Afterward, the required cement amount was gradually added and mixed into the slurry. Finally, the sand was added to the benchtop mixer, and ASTM C305 was followed to complete the mixing process.

After mixing and performing a flow test to determine the mortar's workability, beam specimens of 40 × 40 × 160 mm size were cast according to ASTM C348. Beams were cast in two layers with 12 times tamping in each layer. Following 24 ± 1 h of casting, the beams were unmolded and housed in a controlled chamber in lime water at 23.0 ± 2.0 °C temperature for seven days and 28 days until the testing.

### Mortar testing procedures

#### Flow table tests

**Figure 4:**
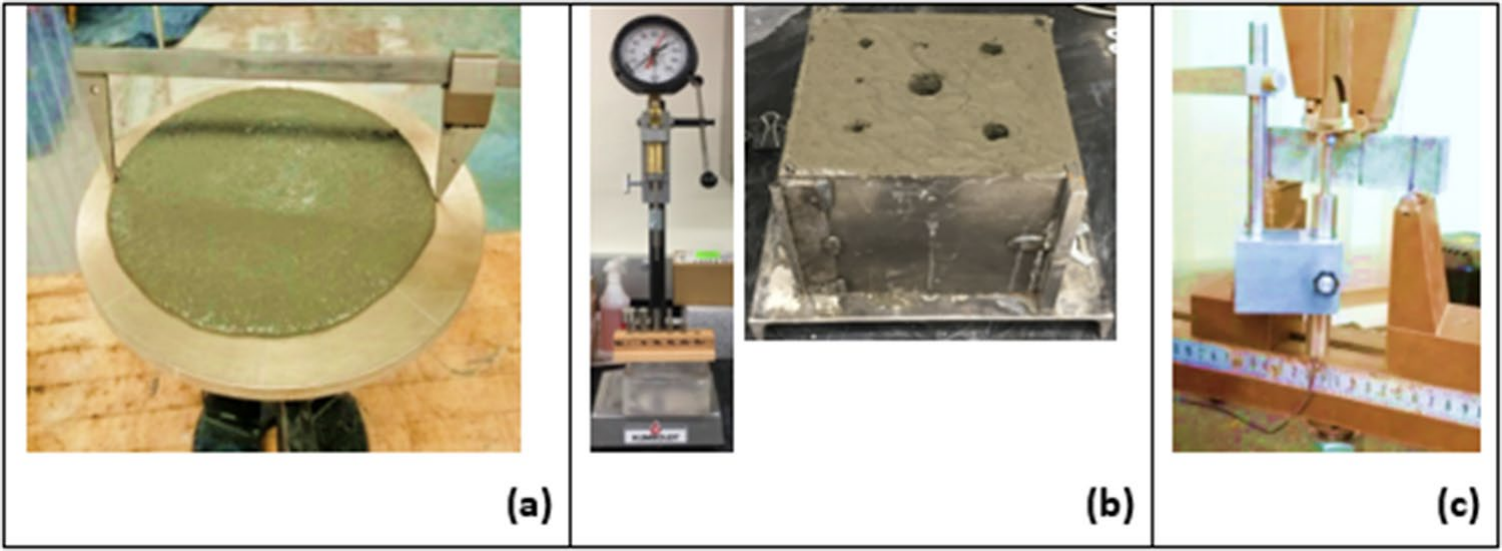
(**a**) Flow table test shown for 0.065 ChNF mixes, (**b**) setting time test by penetration resistance, (**c**) flexural strength test setup in third point loading configuration.

Flow tests were performed on all fresh mortars after mixing according to ASTM C1437 (Fig. 4a). The mold was filled in two lifts with 20 times tamping in each layer to compact sufficiently. The mold was removed, and the flow table was dropped 25 times in 15 s. The mortar diameter along four lines was added and recorded as flow in percentage.

#### Setting time tests

Mortar setting time was evaluated following the ASTM C403 by penetration resistance. The tested dosages of ChNF and ChNC in the mortar were 0%, 0.035 wt%, 0.05%, 0.075% and 0.1%. The carboxylate-based SP with 0.05 wt% dosage was used as a control. The test was performed on 152-mm cubes using the standard penetration needles (Fig. 4b). A regression curve was fitted to the collected penetration resistance data plotted against the elapsed time. The Regression curve points corresponding to the resistance of 3.5 and 28 MPa were the initial and final setting times, respectively.

#### Flexural strength test

After seven and 28 days of curing, beam specimens were tested for flexural strength by the third-point (four-point) loading configuration (Fig. 4c). Before testing the beams, dimensions were measured, and a plate was attached to the beam to facilitate the linear variable differential transformer (LVDT) to measure the mid-point deflection. The test was performed in an 8.9-KN Instron universal testing machine at a 0.0017 mm/s deflection rate. The deflection reading was recorded using an LVDT against load increment to obtain the load–deflection graphs and calculate the fracture energy (area under the graph). The flexural strength was calculated using Eq. (2),

2 $$R=\frac{PL}{b{d}^{2}}$$

where P is the maximum load, L is span length, b is beam width, and d is the beam depth.

#### Compressive strength test

Portions of prisms broken after flexural strength tests were used for compressive strength evaluation after 7 and 28 days of curing following ASTM C349. Two 40-mm plates were placed on both loading surfaces of the test specimens according to the standard requirements. The test was performed in Test Mark universal testing machine, where the load rate was maintained between 900 and 1800 N/s per standard guidance. The crosshead travel was recorded, and a stress–strain curve was obtained, which was used to calculate the modulus of elasticity (E) for each specimen.

#### Total organic carbon (TOC)

Total carbon (TC) and inorganic carbon (IC) were calculated in the mortar powder samples using Shimadzu total organic carbon analyzer (Model TOC-L CSH/CSN) to analyze the presence and dispersion of the ChNMs. Sample holders were cleaned with 2 N hydrochloric acid and rinsed in DI water, then dried and placed in a muffle furnace for 30 min at 900 °C before use. After the samples were placed in the holder, TC was determined by firing at 900 °C. Phosphoric acid was added at 200 °C to the sample to calculate the IC, and the TC was determined by a non-dispersive infrared gas analyzer. The difference between the two readings is the total organic carbon (TOC = TC − IC). TC calibration was done by Dextrose (C6H12O6, 40.0% C, Fisher Scientific catalog# D16-500, lot # 170,457), whereas IC calibration was done using Sodium bicarbonate (NaHCO3, 14.3% C, Fisher Scientific catalog# S233-500, lot# 172,497).

#### TGA and FTIR spectroscopy of mortar

Thermogravimetric analysis (TGA) was performed on the control and ChNM-mortars (0.1 wt% load), and the degree of hydration and percent hydrates were calculated to evaluate the effect of ChNMs. A small mortar batch was produced, kept fully sealed, and then taken out for testing on 7 and 28 days. Following the procedure in reference^35^, the samples were quenched in liquid nitrogen and dried in a freeze drier before the TGA test. The test was performed in a Mettler Toledo TGA analyzer in a nitrogen-controlled environment. The starting temperature was 25 °C, and the test was run up to 1000 °C with a temperature gradient of 20 °C/min. Chemically bound water was calculated from the mass loss between the temperature range of 105–1000 °C subtracting the mass loss due to the decarbonation that occurs between 600 and 780 °C. The normalized chemically bound water per gram of paste sample with respect to the maximum hydration of ordinary cement (0.23 g/g of cement) paste is used to measure the degree of hydration (DOH)^35^. In addition to DOH, hydrates content was determined for 28 days based on the TGA data according to Matos et al.^36^ using Eq. (3), where ${W}_{60^\circ}$C, ${W}_{405^\circ}$C and ${W}_{500^\circ}$C are the weight of the samples at the respective temperature in the subscript.

3 $$\%Hydrates=\frac{{W}_{60^\circ{\text{C}}}-{W}_{405^\circ{\text{C}}}}{{W}_{500^\circ{\text{C}}}}\times 100$$

Fourier-transform infrared spectroscopy (FTIR) analysis was done on specimens from the same batch as TGA to identify any changes in hydration products and matrix compositions based on the intensity of the absorbed infrared radiation by the different functional groups using a Nicolet iS-50, Thermo Fisher Scientific FTIR spectrometer. The spectra under attenuated total reflection (ATR) mode for frequencies were obtained between 4000 and 600 cm^−1^. The resolution was set to 1 cm^−1^, and 64 scans were made for each sample.

#### Nuclear magnetic resonance (NMR)

Solid-state ^29^Si NMR was conducted on ChNM-cement paste samples to analyze the influence of ChNM on the mean chain length of the silicate groups at both 7- and 28-day ages. Cement paste was selected for NMR to eliminate the impact of sand in mortar. The NMR experiment was conducted using a VARIAN DD2 600 MHZ spectrometer with a 4 mm ^29^Si solid-state NMR probe. The spectra were collected at 119.6 Hz frequency, and a single pulse experiment was carried out. The experiment was conducted under magic angle spinning (MAS) at 8000 Hz. A pulse width of5.4 µs and a relaxation delay of 20 s were set as input parameters. A 4-mm zirconia rotor was used to load the powdered sample and insert it into the probe. For each sample, up to 2048 scans were taken. The spectra shift in ppm was referenced with respect to tetramethyl silane (TMS), with the peak at 0 ppm.

^29^Si NMR is used to analyze silicate polymerization, which is the number of bonds silicate tetrahedrons produce with the adjacent tetrahedra. A silicate tetrahedron can be expressed as Q^n^, where *n* is the number of shared oxygen atoms with adjacent tetrahedra. A silicon tetrahedron can share maximum of four oxygen atoms. Hence the value of n should be 0 to 4. The conservative ranges in the chemical shift, as represented by Kim et al., were used to differentiate silicate species^7^. In this study, peaks at 67, 75, and 80 ppm correspond to Q^0^, Q^1^, and Q^2^, respectively. The obtained spectra from ^29^Si NMR were deconvoluted using the Gaussian method in the Origin program. The relative abundance of the silicate species is obtained from the deconvoluted spectra after integration. The computed parameters are polymerization degree (PD), mean chain length (MCL), and degree of hydration (DH), and they are calculated according to Wang et al.^37^

4 $$Polymerization\; degree (PD)=\frac{{Q}^{2}}{{Q}^{1}}$$

5 $$Mean\; chain\;length (MCL)=\frac{2({Q}^{1}+{Q}^{2})}{{Q}^{1}}$$

6 $$Degree\;of\;Hydration (DH)=\frac{{Q}^{1}+{Q}^{2}}{{Q}^{0}+{Q}^{1}+{Q}^{2}}$$

#### X-ray diffraction (XRD) analysis

Powder XRD analysis of the samples was performed using the 2021 Bruker D8 Discover TXS-HE A25 XRD analyzer. The XRD instrument was equipped with a rotating Cu anode (Kα λ = 1.5418 Å), 0.3 × 3 mm cassette tungsten filament, Atlas goniometer, and a UMC 1516 motorized stage. Samples (~ 50 mg) were placed in a low-background sample holder. The sample holder is installed on the xyzϕχ stage of the XRD system and set in place with a laser-video alignment system. An EIGER2 R 500 K detector in 1D max 2θ mode with a 78 × 25 mm panoramic axial Soller (2.5°) positioned at a 285.4 mm sample-detector distance. Collection of individual XRD tracings from 5° to –50° 2θ was made for 486 s in coupled 2θ/θ mode with power settings of 45 kV and 120 mA, X–Y grid scanning, and continuous stage rotation (ϕ). On the source side, the TXS-HE beam passed through a focusing Goebel mirror, a 1.0 mm divergence slit, and a 2.5° axial Soller, with a fixed source-sample distance of 425 mm. Initially, images were processed with Bruker DIFFRAC.EVA before importing into MDI JADE XRD software to obtain peak positions and intensities and identify minerals using the International Centre for Diffraction Data (ICDD) powder diffraction file (PDF) database. Reported phases are identified below with their ICDD PDF# specified in parentheses.

## Results and discussion

### Chitin nanomaterials characterization

**Figure 5:**
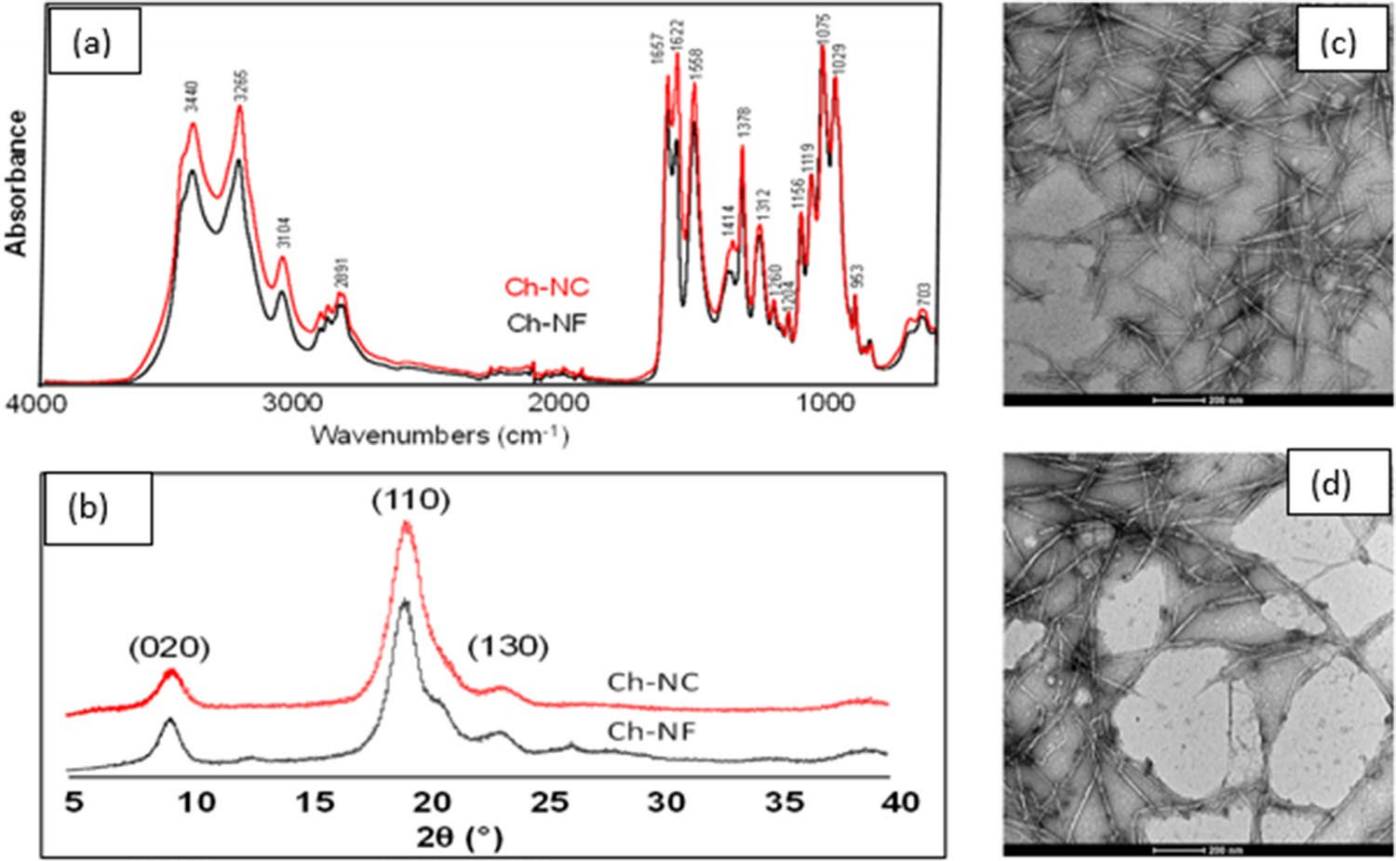
(**a**) FTIR spectra of the two ChNMs, (**b**) XRD spectra of ChNMs, (**c**) example TEM images of ChNC, and (**d**) ChNF.

From the FTIR spectra of ChNC and ChNF (Fig. 5a,b), two strong bands at 1657 cm^−1^ and 1558 cm^−1^ were assigned to the acetamide group. The band at 1624 cm^−1^ is a distinctive signature band of α-chitin. ChNC shows relatively higher absorbance intensity at 1622 cm^−1^ and 1414 cm^−1^ than ChNF due to the oxidation of C6-hydroxyl groups to carboxylate groups during the TEMPO-Oxidation procedure. ChNC also shows higher intensity vibrational stretching modes of surface OH groups (3440 cm^−1^) and NH_2_ (3265 cm^−1^), likely due to the higher surface area of ChNC.

The size and morphological characteristics of ChNMs were identified from TEM image analysis (Fig. 5c,d). ChNC shows rod/whisker shapes with an average length of 211 nm, a width of 8.7 nm, and an average aspect ratio of 24. The mechanically processed ChNFs show a fiber-like structure with a larger width (16 nm), length (1068 nm), and aspect ratio (67) compared to ChNCs. Based on the XRD analysis, the crystallinity index of ChNC and ChNF was found to be 92%, which is a little higher than the original chitin indicating that ChNMs sustained the original crystalline structure of the chitin source.

The ς-potentials were found to be -56.1 ± 4.5 (at pH = 7.6) for ChNC and + 3.9 ± 0.7 (at pH = 6.9) for ChNF in a neutral pH environment. The high negative values of ChNC indicate the existence of carboxylate ions, and small positive values for ChNF are due to the ammonium ions present on its surface. From the conductimetric titration, the surface charge density of the [-COOH] group was found to be 0.36 mmol for ChNC and 0.01 mmol for ChNF, which indicates that TEMPO-oxidized ChNC has a higher concentration of surface charge groups compared to the mechanically fibrillated ChNF. The characteristic of ChNM's is summarized in Table 3.

**Table 3:** Summary of characterization results for ChNC/ChNF.

Properties	ChNC	ChNF
Shape	Rod/whisker	Fibrillous
Size	Width 8.7 ± 4 nm	Width 16 ± 10 nm
	Length 211 ± 80 nm	Length 1068 ± 765 nm
Aspect ratio	24 ± 20	67 ± 90
**Zeta potential**		
In DI water	− 56.1 ± 4.5 mV (at pH = 7.6)	+ 3.9 ± 0.7 (at pH = 6.9)
In pore solution	− 28.04 ± 2.6 mV (pH = 12.71)	− 24.02 ± 9.1 mV (pH = 12.71)
Surface groups (COOH)	0.36 mmol	0.01 mmol

### Fresh and hardened characteristics of mortar composites

#### Workability

The workability of the fresh mortars was characterized by the flow table test, and the flow values in percentage are tabulated in Table 4**.** It is seen that the control had the highest flow numbers. The addition of both ChNC and ChNF reduces the workability of the fresh mortar. For ChNC, the flow number decreases with the increase in the concentration of ChNC. On the other hand, no definite trend with concentration was observed for flow values of ChNF-mortars. For 0.1% ChNC, the flow number was reduced by a maximum of 26%. For ChNF, the maximum reduction of 17% was recorded for 0.035% ChNF. For 0.1% ChNF, the reduction in flow value was 11% compared to the control. For 0.05% and 0.1%, ChNF shows higher workability compared to the same doses of ChNC. Overall, the addition of ChNM reduces workability compared to the control mix, likely due to their high specific surface area, hydroxyl, and carboxyl group present in the surface. The free hydroxyl group present on the surface of ChNM's might interact with the cement, water, and cement hydration product through the hydrogen bond, reducing fresh concrete's workability^38^. Moreover, a high number of carboxylate groups may create a flocculating effect because different parts of the chitin molecule connect with varying cement particles, reducing the workability of fresh concrete as was observed with chitosan-cement mortar composites in another study^12^. This effect is expected to be more pronounced in chitin nanomaterials because of their higher specific surface area^12^.

**Table 4:** Flow number, compressive strength, and elastic modulus of mortar at 7 and 28 days.

Mixture ID/tested property	Flow number (%)	Average compressive strength (MPa) (SD)		Average modulus of elasticity relative to control	
		7D	28D	7D	28D
Control	124	32 (3.30)	52 (4.39)	1.00	1.00
0.020ChNC	117	34 (1.28)	53 (1.54)	1.09	1.91
0.035ChNC	109	35 (0.81)	53 (1.88)	1.69	1.34
0.045ChNC	111	32 (1.41)	52 (2.55)	1.51	0.79
0.050ChNC	105	32 (1.22)	51 (1.94)	*	1.24
0.10ChNC	92	33 (3.26)	48 (3.40)	1.20	0.80
0.035ChNF	102	32 (1.37)	52 (2.13)	1.69	0.82
0.045ChNF	113	31 (1.23)	51 (2.35)	1.23	1.43
0.050ChNF	109	32 (2.19)	51 (2.37)	1.06	1.14
0.055ChNF	111	31 (0.90)	50 (1.62)	1.67	1.42
0.065ChNF	108	32 (1.16)	53 (2.20)	1.51	1.43
0.10ChNF	110	33 (3.26)	46 (2.25)	1.10	0.98
0.05ChNC + 0.05ChNF	111	33 (1.30)	51 (2.18)	1.16	0.99
0.1SP	133	34 (0.95)	55 (2.03)	2.00	1.36
0.1ChNC + 0.1SP	124	40 (1.66)	51 (1.47)	1.62	1.54
0.1ChNF + 0.1SP	126	40 (1.58)	55 (1.38)	1.71	1.60

*Data not recorded due to instrument error.

#### Setting time

**Figure 6:**
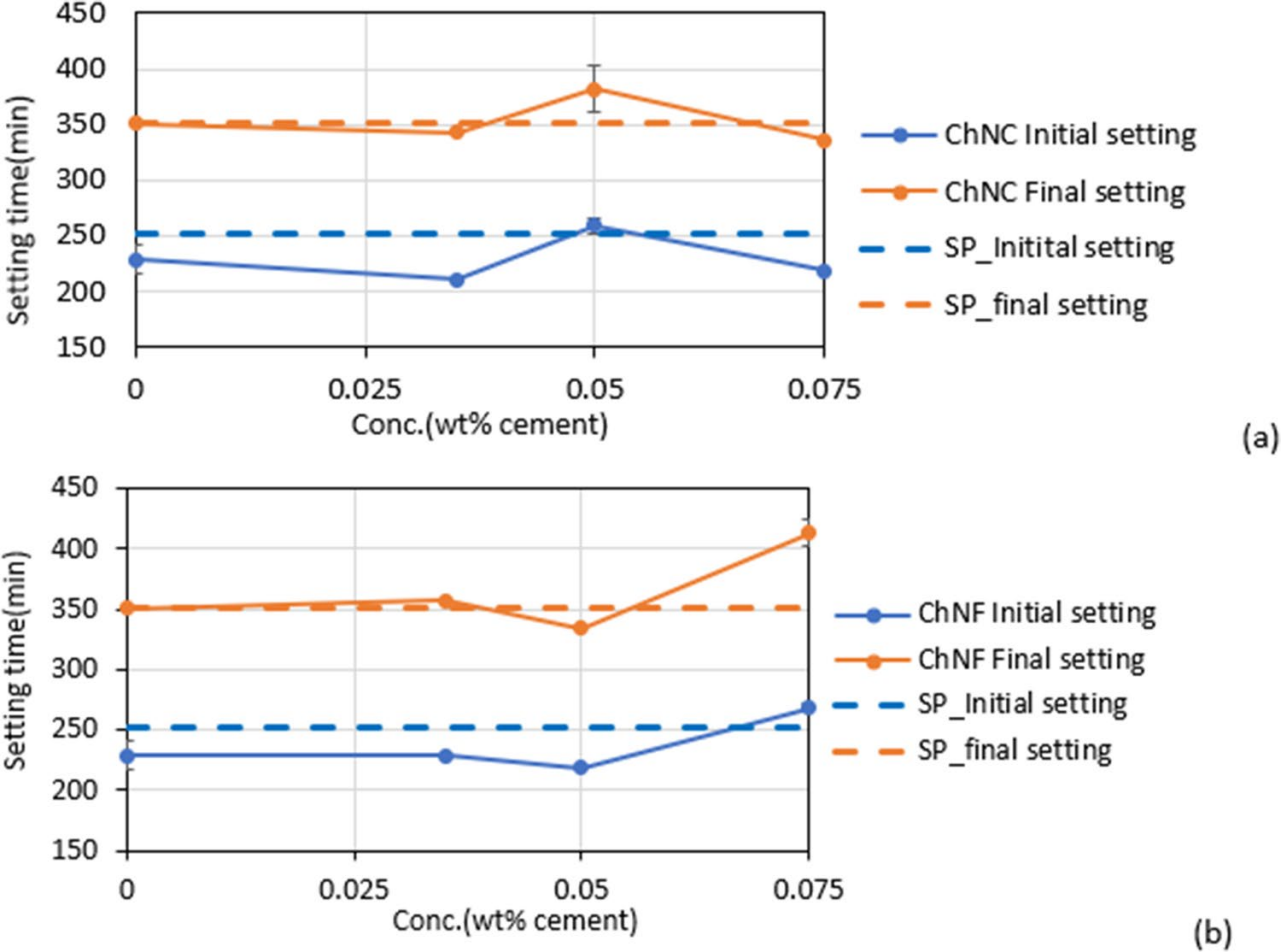
Initial and final setting times for (**a**) ChNC-mortars and (**b**) ChNF-mortars.

The initial and final setting times of the mortar obtained from the penetration resistance test are shown in Fig. 6. The mass ratio of cement:sand:water was 1:2.5:0.52. The initial and final setting time of the mortar with SP on a concentration of 0.05 wt% based on dry cement mass is shown as dashed lines in Fig. 6. The results show that mortar with 0.05 wt% ChNCs has the longest setting time compared to mortars with higher and lower concentrations of ChNC. The initial setting time is similar for 0.05 wt% ChNC-mortar and the control, but the final setting time is delayed by approximately 30 min. In the case of ChNF-mortars, 0.075 wt% ChNF shows similar or longer setting times than SP mortar. The initial setting time for 0.075 wt% ChNF-mortar is nearly identical to the control, but like with ChNCs, the final setting time is delayed by 50 min with 0.075 wt% ChNF.

As shown earlier in Table 3, high negative ς-potentials of − 28.04 ± 2.6 mV and − 24.02 ± 9.1 mV were measured for ChNC and ChNF in a simulated cement pore solution. With these large anionic surface charges, ChNCs and ChNFs are expected to adsorb on the positively charged clinkers, disperse them by electrostatic repulsion and delay the setting time. However, this effect depends on the concentration of the ChNC and ChNF.

#### Compressive strength

The average of six tested specimens per mortar mixture and the associated standard deviation in compressive strengths (*f'*_*c*_) at 7 and 28 days are shown in Table 4. A statistical t-test (with an alpha value of 0.05) was used to assess the statistical significance of the change with ChNMs compared to control. As expected, the compressive strength increased with a curing time of 7–28 days for all tested mortars. However, the compressive strength results do not show any trends with the concentration of ChNMs at 7 or 28 days.

For ChNC, at 7 days, the greatest improvement of ~ 9% was with 0.035 wt% dose (*p* value = 0.023 < 0.05). At 28 days, the maximum enhancement of 2.5% was observed with 0.02% ChNC (*p* value  = 0.004 < 0.05). For ChNF at 7-days, the maximum increase was ~ 4% for 0.1% ChNF (*p* value  = 0.049 < 0.05). The hybrid blend of 0.05 wt% ChNC + 0.05 wt% ChNF did not significantly impact the mortar at 7 and 28 days. Under compression, ChNMs may act only as stiff fillers and offer slight *f'*_*c*_ enhancement. Their greater contribution is expected under tension, as will be discussed in flexural strength results.

Additionally, the addition of SP improved 7 and 28 days *f'*_*c*_ compared to the control (mortar without SP), which could be attributed to better workability and, thus, denser packing. In fact, the greatest increase in *f'*_*c*_ among all the tested mortars was observed for 0.1 wt% ChNF + 0.1 wt% SP with an increase of ~ 25% and 7% at 7 and 28 days (*p* value = 0 < 0.05).

#### Modulus of elasticity

The modulus of elasticity computed from the stress–strain curves obtained during the compression tests is shown in Table 4 in the form of relative values to the modulus of the control. Improvements were noticed for all doses of ChNC/ChNF at 7 days. For both ChNC and ChNF, a maximum 69% improvement was observed at 0.035 wt% at 7 days. At 28 days, the highest enhancement in modulus was 91% compared to the control with 0.02 wt% ChNC followed by 34% with 0.035 wt% ChNC. For ChNF, the greatest improvement was 43% with 0.045 wt% and 0.065 wt% concentration, followed by 0.055 wt% with an improvement of 42% over the control. No trend is observed between the modulus and dose of ChNC and ChNF on either 7- and 28-day aging periods. Though 0.1 wt% ChNC and ChNF decreased the modulus at 28 days, adding 0.1 wt%, SP improved the modulus of elasticity by about 54% and 60%, respectively. Thus SP shows excellent synergy with the studied ChNMs. The reason for improved modulus in the mortar with ChNC and ChNF might be the higher modulus and crystallinity of ChNMs than in the paste matrix.

#### Flexural strength

**Figure 7:**
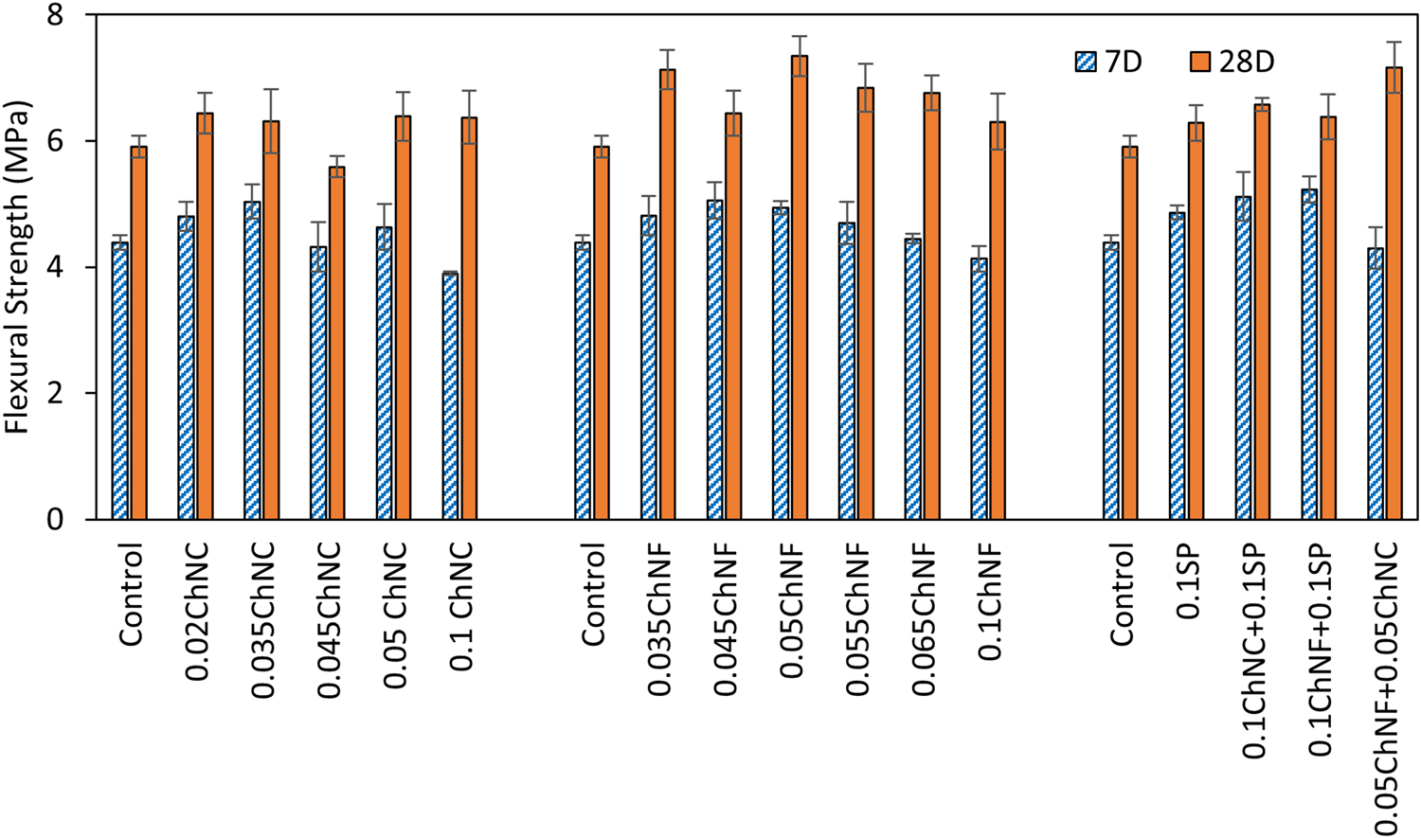
Impact of ChNC and ChNF additions on 7- and 28-days flexural strength of mortar.

The flexural strength of mortar formulations with ChNC and ChNF is shown in Fig. 7. Most of the doses of ChNC/ChNF improved the flexural strength at 7 days except 0.1 wt%, and all doses increased flexural strength at 28 days. For ChNC, the maximum increase of 15% was found for 0.035 wt% (*p* value  = 0.0007) at 7 days followed by 0.02 wt% with an improvement of 10% (*p* value  = 0.012 < 0.05). At 7 days, the flexural strength of 0.1 wt% ChNC dropped by 11%, which might be caused by the poor workability of the mix as indicated by the flow values of the fresh mix. At 28 days, all doses of ChNC have comparable improvement between 7 ~ 9%. The greatest improvement in flexural strength at 28 days for ChNC mortars was for 0.1 wt% ChNC with 0.1 wt% SP. In Fig. 6, the flexural strength of different doses of ChNF shows bell-shaped curves for both 7 and 28 days. At 7 days, 0.045 wt% shows the highest increase (15%) in flexural strength, followed by 0.05 wt% of ChNF; both improvements are statistically significant based on the *p* value results of the t-test. Flexural strength development is more prominent on 28 days than the 7 days with ChNF over the control. A maximum improvement of 24% was seen at this age for 0.05 wt% ChNF. With higher doses beyond the optimum amount, ChNF might entangle, causing possible voids and zone of stress concentrations which can reduce the strength. From the flexural strength results, it is seen that for equal doses, ChNF performed better than ChNC.

Similar to the behavior reported for carbon nanotubes in another study^33^, ChNMs have a filler role under mechanical loading in compression, but a more significant enhancement of the composite strength is seen under tension (and similarly flexural loading). During typical flexural strength tests on cement, nano cracks initiate under loading in the tension region. The possible reason for improving the flexural strength with ChNC and ChNF could be the bridging of nano cracks and pores, with a resulting delay in crack propagation before the peak load is reached. Better flexural performances with ChNF over ChNC for the same dose may be attributed to the longer length, higher aspect ratios, and fiber-like structure of ChNFs.

#### Fracture energy (area under the flexural load–deflection graph)

**Figure 8:**
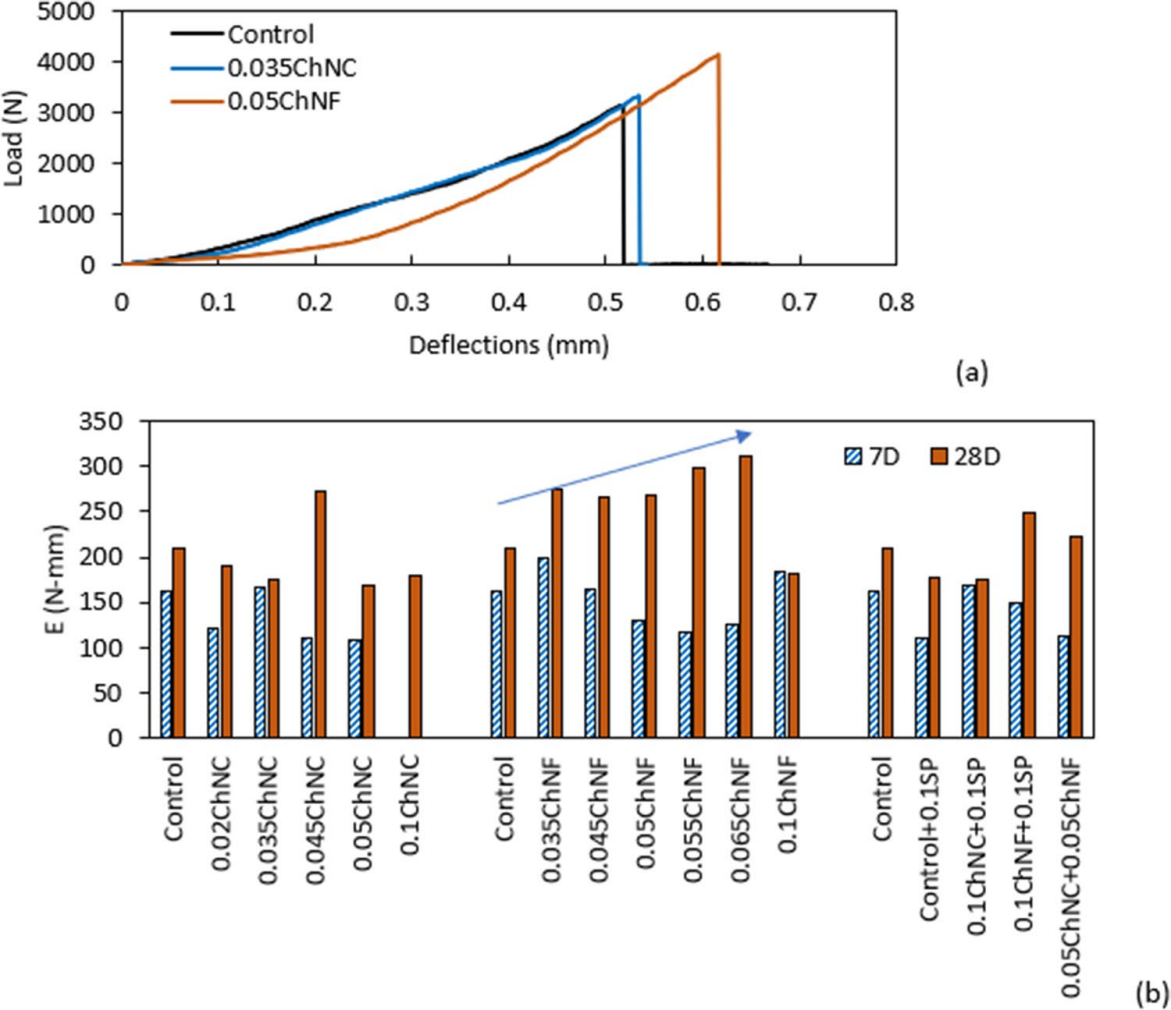
(**a**) Select load–deflection graph for ChNC and ChNF at 28 days, (**b**) computed average fracture energy at 7 and 28 days with ChNC and ChNF (data for 0.1ChNC at 7 days not available).

The load–deflection graph of one representative mix from ChNC and CHNF and the control mortars are shown in Fig. 8a. The fracture energy of the mortar mixes calculated based on the load–deflection graphs from the flexural strength test of all mixes is shown in Fig. 8b. ChNC did not show any positive effect on the fracture energy of the mortar, neither at 7 days nor at 28 days, except for 0.045 wt% at 28 days. Contrary to ChNC, ChNF showed improvements in fracture energy at both 7 and 28 days. At 7 days, 0.035 wt% ChNF improved the fracture energy by 23%, followed by an improvement of 14% with 0.1 wt% ChNF. At 28 days, the maximum improvement in fracture energy was 49% for 0.065 wt% ChNF, followed by a gain of 43% for 0.055 wt% ChNF. At the same age, 0.035 ChNF and 0.05 ChNF improved the fracture energy by 31% and 28%, respectively. Though the fracture energy of the hybrid mix and the SP mix did not improve at 7 days, improvements of 20% and 7% were found for hybrid and 0.1ChNF + 0.1SP, respectively, at 28 days. The improvement in fracture energy with ChNF might be due to their fiber-like structure and longer dimension, allowing transferring load across cracks.

#### Total organic carbon (TOC)

The organic carbon content was evaluated for three representative samples from various locations of the bottom portion of the beams after the flexural test, and the result is tabulated in Table 5. From the results, chitin-mortar samples possess significantly more organic carbon compared to the control, which demonstrates that ChNMs were present in the beams' fracture surface and ChNM's were dispersed well in the beam supporting the improvements seen in flexural strength.

**Table 5:** Total organic %Carbon in the specimens from the broken beam faces.

Mix	Total organic %Carbon
Control	0.0027
0.05ChNC	0.0607
0.05ChNF	0.1233

#### Thermogravimetric analysis (TGA) of mortar

**Figure 9:**
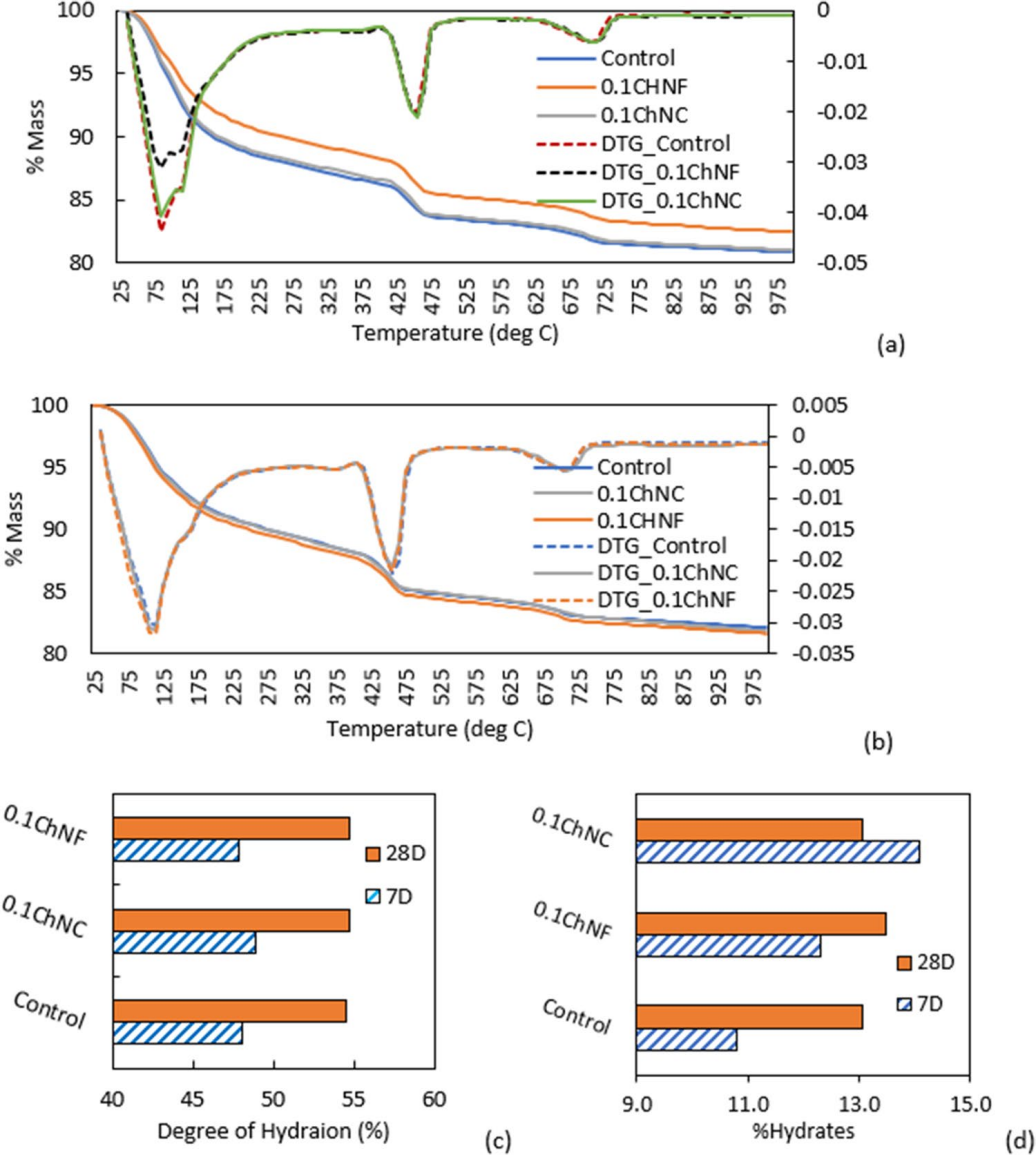
TGA of control, ChNC, and ChNF mortars at (**a**) 7 days, (**b**) 28 days and (**c**) summary of computed 7- and 28-day degree of hydration (**d**) % hydrates at 28 days based on TGA results.

TGA was performed on samples from the control and 0.1 wt% ChNC and ChNF (highest dosage) at 7- and 28-days ages. The initial %mass loss vs. temperature and 1st derivatives are shown in Fig. 9a,b. The computed degree of hydration and %hydrates from TGA is shown in Fig. 9c,d, as described previously in the Materials and Methods section.

The amount of bound water was ~ 13% in control and ChNC and ChNF at 7 days. At 28 days, the bound water was around 14% for control, 0.1 wt% ChNC, and 0.1 wt% ChNF, respectively. The degree of hydration (DOH) was calculated from the TGA data, as described in the Materials and Methods section. At 7 days, ChNC-mortar slightly improved DOH by 1.75%. ChNF did not affect DOH at this age. At 28 days, ChNC and ChNF show no obvious improvement in DOH (only 0.39% and 0.25%, respectively).

Additionally, the hydrates content was calculated for 7 and 28 days based on the TGA data using Eq. (2), and the results are shown in Fig. 9d. Based on the figure, ChNF-mortar contains 14% and 3.3% more hydrates compared to control at 7 and 28 days, respectively. For ChNC-mortar, though at 7 days hydrates amount was 30% higher than control, at 28 days the hydrate content was the same as the control. These results imply that ChNMs-mortar created higher %hydrates; however, they did not significantly affect DOH characterized based on TGA of aged mortar samples.

#### Fourier transform infrared spectroscopy (FTIR) and X-ray diffraction (XRD) of mortar

**Figure 10:**
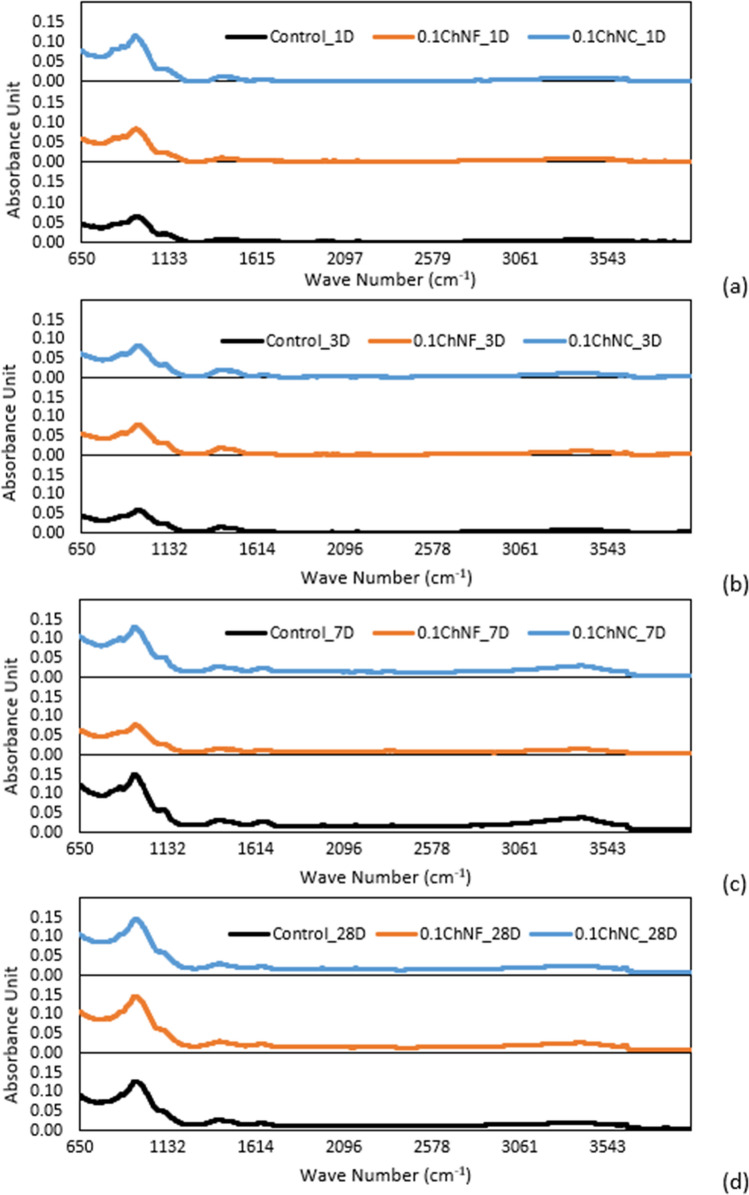
FTIR Spectra of control, 0.1 wt% ChNC and ChNF mortars at (**a**) 1 days, (**b**) 3 days, (**c**) 7 days and (**d**) 28 days.

The FTIR spectra of the control and 0.1 wt% ChNC and ChNF at 1, 3, 7, and 28 days are shown in Fig. 10. The H–OH stretching band at 1650 cm^−1^ and 3000–3500 cm^−1^ represent the water in the matrix^39^. The band at 900 ~ 1100 cm^−1^ is a Si–O asymmetric stretch which mainly represents the early stage of calcium-silicate-hydrates (C–S–H)^40^.

For all the tested mortar mixtures, the peak around 948 cm^−1^ on 1 day shifted to the higher wavenumbers on 7-day and moved slightly further to a higher wavenumber on 28 days, indicating the polymerization of silicate tetrahedra with time due to the hydration process. For the control, at 1 day, the peak for Si–O asymmetric stretch was 948.53 cm^−1^. The corresponding peak for ChNF and ChNC-mortars was at 946.41 cm^−1^, suggesting that control has higher silicate polymerization after 1 day. However, the shift of this peak is slightly higher for ChNC and ChNF over the control at 7 and 28 days, which signifies that ChNM's promote the silicate polymerization at later curing times than the control. As curing time increased, the peaks around 3600 cm^−1^ were broadened and with higher intensity for all three mixes, indicating more OH groups present due to the production of portlandite during the hydration reactions. At 7 days, ChNC- and ChNF-mortars showed a lower absorption stretching vibrational band around 3400 cm^−1^ which corresponds to the bonded OH group and free water, and it is attributed to a reduction of portlandite with a simultaneous increase in C–S–H content^41^, TGA also confirmed higher %hydrates with ChNMs in the cement mortar (Fig. 9d). However, at 28 days, the relative (to control) change in the absorption value of the stretching band around 3400 cm^−1^ was not significant. A band at 2350 cm^−1^ was visible for ChNC- and ChNF-mortars and was not present for the control. This peak is CO_2_ from the environment and not relevant to the samples.

**Figure 11:**
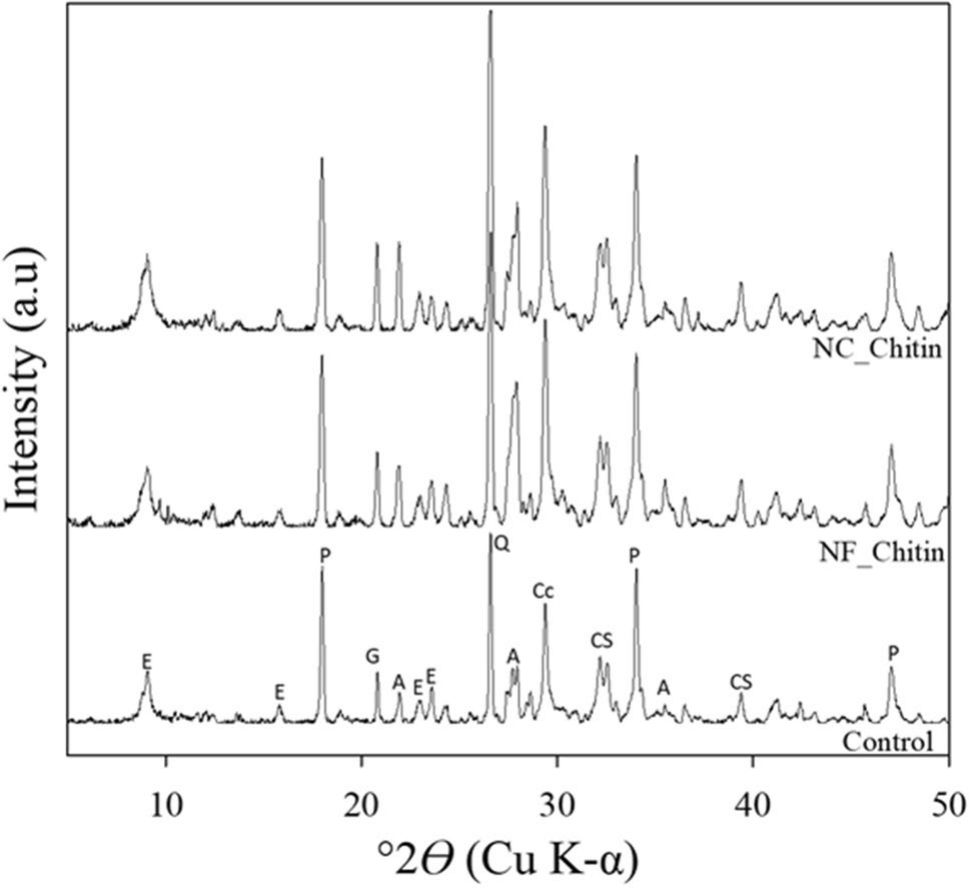
XRD patterns for cement samples with offset intensities. See text for specific powder diffraction file designations. E: ettringite, P: portlandite, G: gypsum, A: anorthite-like Ca-Al silicate, Q: quartz, Cc: calcite, CS, calcium silicate (including larnite).

The same results as FTIR were seen based on XRD spectra of aged samples (older than 28 days) taken from fracture faces of tested beams. X-ray diffraction analysis in Fig. 11 indicated that the samples were primarily composed of quartz (SiO2, 033-1161), portlandite [Ca(OH)_2_, 044-1481], calcite (CaCO_3_, 04-023-8700), ettringite [Ca_6_Al_2_(SO_4_)_3_(OH)_12_·26H_2_O, 1-1451], calcium silicates, including Ca_3_SiO_3_ (055-0740) and larnite (Ca_2_SiO_4_, 33·0302), an anorthite-like Ca–Al silicate (CaAl_2_Si_2_O_8_, 12·0301), and gypsum (CaSO_4_·2H_2_O, 033-0311).

Based on the consistent XRD peak intensities of control, ChNM-mortars, the samples contained similar quantities of ettringite, portlandite, and calcite.

#### NMR analysis of cement paste

The relative abundance of the different silicate species and the calculated parameters from the deconvoluted spectra for all the tested specimens of ChNM-cement paste at 7 and 28-day are tabulated in Table 6. From the deconvoluted spectra, no Q^3^ and Q^4^ were evident, which means there is no cross-linking between the chains of silicates. The tricalcium silicate (C_3_S) present in the cement primarily consist of silicate monomers with no chain and branching. With the advances in the hydration reaction, polymerization occurs, and Q^0^ converts to Q^1^ or higher-order linkages. The relative abundance of Q^1^ and Q^2^ increased for ChNF-paste compared to the control at both 7 and 28 days. Similarly, the relative abundance of Q^1^ and Q^2^ for ChNC-paste increased after 28 days. However, the degree of polymerization for ChNC-pastes did not improve with respect to control at 7-day.

**Table 6:** Percentage of the silicate species and parameters from 29Si NMR at 7 and 28-day.

Mix	Q^0^ (~ 67) (%)	Q^1^(~ 75) (%)	Q^2^(~ 80) (%)	PD	MCL	DOH
**7-days**						
Control	46	44	10	0.23	2.45	0.54
0.05ChNF	40	48	11	0.24	2.47	0.60
0.05ChNC	49	42	9	0.21	2.41	0.51
**28-days**						
Control	39	47	13	0.28	2.56	0.61
0.05ChNF	30	50	20	0.39	2.79	0.70
0.05ChNC	35	52	13	0.25	2.50	0.65

**Figure 12:**
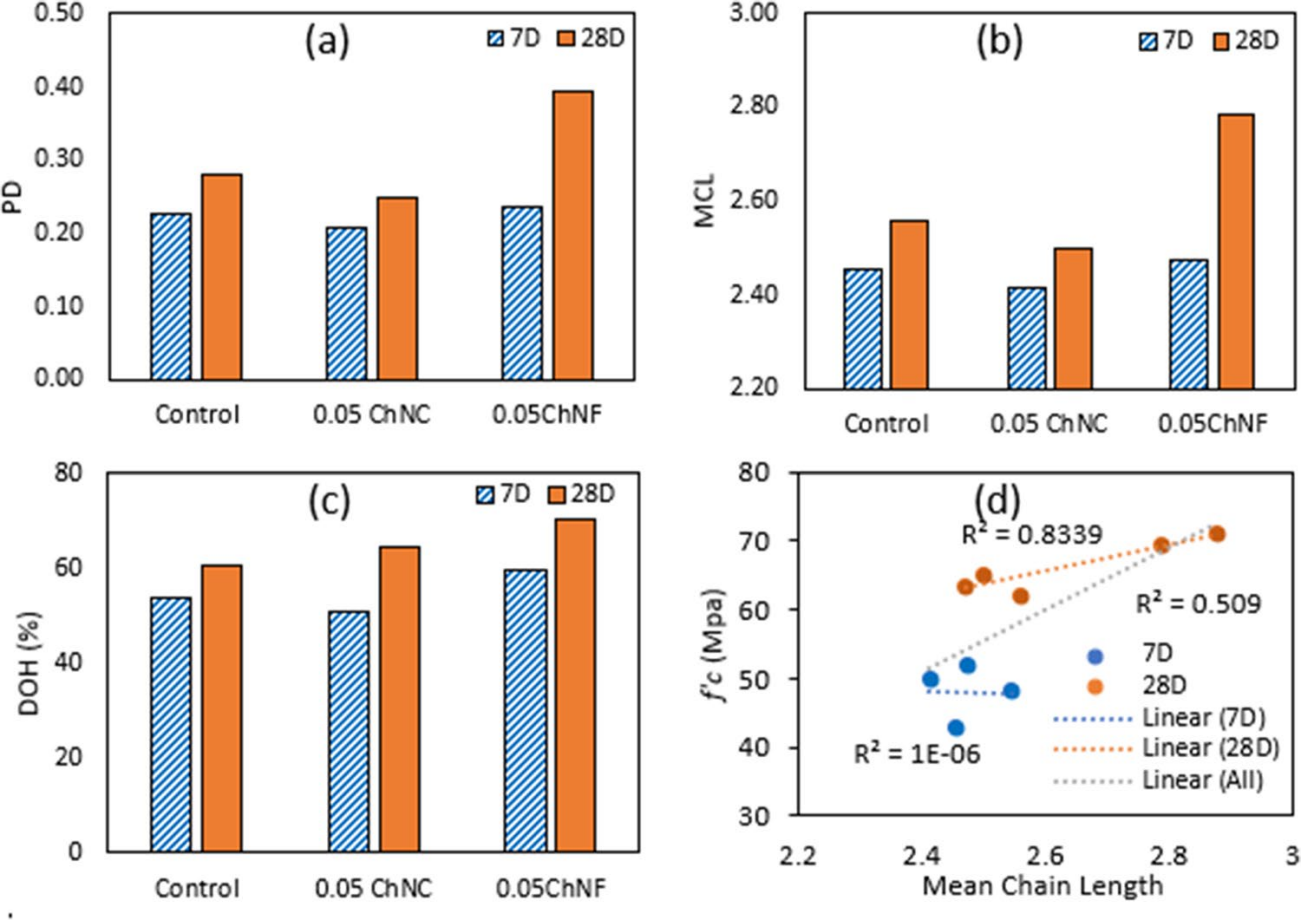
Analysis of ^29^Si NMR spectra for ChNM-cement paste at 7 and 28 days (**a**) polymerization degree, (**b**) mean chain length, (**c**) degree of hydration and (**d**) correlation of mean chain length (MCL) and compressive strength (*f'*_*c*_).

The parameters calculated from ^29^Si NMR for 0.05 wt% ChNC and ChNF cement paste at 7 and 28 days are compared in Fig. 12. Polymerization degree, MCL, and DOH were improved with 0.05 wt% ChNF at 7 and 28-day. The 28-day improvements in the parameters were 41%, 9%, and 16%, respectively, compared to the control. For ChNC, DOH improved at 28-day by 7%. The increase may be attributed to functional groups in ChNM's surface bonding with the cement hydration products. For example, the surface OH group in ChNM's might form Si–OH, which may mutually connect and condense into higher polymerization^42^. The mean chain length calculated from NMR and the compressive strength for the same mixtures for both 7 and 28-days are plotted in (d). The 7-day strength did not show a strong correlation as the increase in MCL was less prominent at 7-days with ChNMs. The 28-days strength had a strong correlation with MCL (R^2^ = 0.83).

## Conclusions

The prospect of two distinctive ChNMs to enhance the mechanical properties of a mortar formulation was assessed. These two chitin nanomaterials (ChNMs) were produced by two fundamentally different processes from the same raw material derived from shrimp shell waste and characterized fully before adding to the mortar system. ChNC and ChNF were different in terms of surface functional groups and morphology and, as a result, were expected to perform somewhat differently in the mortar system.

Mechanical testing of the mortar system indicated that, although ChNMs with SP and few doses of ChNMs improved compressive strength, most doses did not significantly influence the compressive strength. However, the improvement in flexural strength was prominent. The addition of mechanically fibrillated ChNF improved flexural strength by up to 24%. The optimum dose for ChNF was found to be 0.05 wt%. For ChNC, the optimum dose was lower (0.035 wt%) than ChNF, with a maximum improvement of 9% over control. A significant improvement (by up to 91%) in modulus of elasticity was also observed with both ChNC and ChNF. Toral Organic Carbon (TOC) showed significant amounts of organic carbon from chitin present in ChNM samples retrieved from the bottom portion of fracture faces after flexural testing in the tension zone.

Results from flexural strength and fracture energy of the mortars suggest that ChNMs may bridge nano/micropores and delay crack growth. This effect is more pronounced in ChNF due to their higher length and aspect ratio. From TGA analysis, ChNMs had higher %hydrates, though no significant improvement was observed in the degree of hydration. FTIR shows more silicates and less amount of portlandite with ChNMs. However, ^29^Si NMR showed improvements in silicate polymerization, mainly for 0.05 wt% ChNF, which yielded the highest mechanical performance in flexural strength and fracture energy. The mean silica chain also showed a strong correlation with 28-day compressive strength.

In summary, chitin-based nanomaterials can effectively improve the flexural strength and modulus of elasticity of mortar systems with improvement in comprehensive strengths in certain doses and do not reduce the compressive strength in other doses. Chitin nanomaterials showed a good synergy with commercial superplasticizer admixture, further enhancing the 28-day compressive strength by 25%.

The team is performing more investigations to understand the fundamental interactions and mechanisms of ChNMs with concrete components to scale up from mortar to concrete material. Furthermore, we are studying the impact of ChNMs on the microstructure and durability of cementitious systems.

## Data Availability

Some of the additional test data can be made available upon reasonable request. Chitin nanomaterials are available in only finite amounts and are being used for the next round of experiments by the authors. There may be small amounts to be made available upon reasonable request if available after the experiment. However, more details can be made available to produce chitin nanomaterials following the process in this study. Please email the corresponding author for any requests.
