# Author Correction: Impact of chitin nanofibers and nanocrystals from waste shrimp shells on mechanical properties, setting time, and late-age hydration of mortar

**DOI:** 10.1038/s41598-023-50032-4

**Published:** 2023-12-21

**Authors:** Md. Mostofa Haider, Guoqing Jian, Hui Li, Quin R. S. Miller, Michael Wolcott, Carlos Fernandez, Somayeh Nassiri

**Affiliations:** 1 Department of Civil and Environmental Engineering, Washington State University, Pullman, WA 99163 USA; 2 Pacific Northwest National Laboratory, Richland, WA 99352 USA; 3 Composite Materials and Engineering Center, Washington State University, Pullman, WA 99163 USA; 4 Department of Civil and Environmental Engineering, Washington State University, Pullman, WA 99164 USA

Correction to: *Scientific Reports* 10.1038/s41598-022-24366-4, published online 29 November 2022

Quin R. S. Miller was omitted from the author list in the original version of this Article.

The Author Contributions section now reads:

“The authors confirm contribution to the paper as follows: study conception and design: S.N., C.F., M.W., H.L.; data collection: Md.M.H., G.J., H.L., Q.R.S.M; analysis and interpretation of results: Md.M.H., S.N., H.L., Q.R.S.M; draft manuscript preparation: Md.M.H., S.N., H.L., M.W. All authors reviewed the results and approved the final version of the manuscript.”

The original Article has been corrected.

